# HULC: an oncogenic long non‐coding RNA in human cancer

**DOI:** 10.1111/jcmm.12956

**Published:** 2016-10-25

**Authors:** Xin Yu, Heyi Zheng, Matthew T.V. Chan, William Ka Kei Wu

**Affiliations:** ^1^ Department of DermatologyPeking Union Medical College HospitalChinese Academy of Medical Sciences and Peking Union Medical CollegeBeijingChina; ^2^ Department of Anaesthesia and Intensive CareThe Chinese University of Hong KongHong KongChina; ^3^ State Key Laboratory of Digestive DiseaseLKS Institute of Health SciencesThe Chinese University of Hong KongHong KongChina

**Keywords:** long non‐coding RNAs, HULC, cancer, oncogene, prognosis

## Abstract

Highly up‐regulated in liver cancer (HULC) was originally identified as the most overexpressed long non‐coding RNA in hepatocellular carcinoma. Since its discovery, the aberrant up‐regulation of HULC has been demonstrated in other cancer types, including gastric cancer, pancreatic cancer, osteosarcoma and hepatic metastasis of colorectal cancer. Recent discoveries have also shed new light on the upstream molecular mechanisms underlying HULC deregulation. As an oncogene, HULC promotes tumorigenesis by regulating multiple pathways, such as down‐regulation of EEF1E1, promotion of abnormal lipid metabolism, and up‐regulation of sphingosine kinase 1. Pertinent to clinical practice, a genetic variant in the *HULC* gene has been found to alter the risk for hepatocellular carcinoma and oesophageal cancer, whereas cancer patients with high or low expression of HULC exhibit different clinical outcome. These findings highlighted the pathogenic role and clinical utility of HULC in human cancers. Further efforts are warranted to promote the development of HULC‐directed therapeutics.

## Introduction

Only 2% of the human genome encodes protein‐coding genes, whereas the function of the remaining is still poorly defined. With the completion of the Encyclopedia of DNA Elements (ENCODE) project 1, it is now known that a significant portion of this genomic dark matter is transcribed into non‐coding RNAs, which have diverse biological functions 2. Long non‐coding RNAs (lncRNAs) are non‐protein‐coding RNAs with more than 200 nucleotides in length. LncRNAs play a crucial role in the regulation of gene expression and participate in many biological processes, including epigenetics 3, alternative splicing 4, ‘sponging’ small RNAs 5 and translational regulation 6. It therefore comes as no surprise that altered lncRNA expression is implicated in many human diseases, including diabetes 7, infection 8, autoimmune diseases 9 and particularly cancer. In this connection, accumulating evidence have demonstrated the functional involvement of lncRNAs in the pathogenesis of different types of cancer, such as gastric 10, colon 11, lung 12 and pancreatic 13 cancers as well as glioma 14, melanoma 15 and hepatocellular carcinoma (HCC) 16, 17.

Highly up‐regulated in liver cancer (HULC) was originally identified as the most overexpressed lncRNA in human HCC by Panzitt and colleagues in 2007 18. *HULC* gene is located on chromosome 6p24.3 with approximately 500 nucleotides in length and contains two exons. The transcribed RNA lacks substantial open‐reading frame and does not give rise to any protein. Since then, the aberrant up‐regulation of HULC has been discovered in other cancer types 19, 20, 21, 22. To this end, functional characterization indicated that HULC could promote different pro‐tumorigenic phenotypes, such as cell survival, proliferation and invasion 20 *in vitro* as well as tumour growth 23 and angiogenesis 24 *in vivo*. These studies collectively indicate that HULC dysregulation plays a key role in tumorigenesis.

In this review, we examine current evidence regarding the deregulation of HULC in human cancers and its associated mechanisms. Importantly, we discuss the clinical utilities of HULC as disease susceptibility and prognostic markers as well as the possible directions of future investigation.

## Aberrant up‐regulation of HULC in human cancers

Highly up‐regulated in liver cancer is aberrantly up‐regulated in a wide spectrum of human cancers, including hepatocellular carcinoma 18, gastric cancer 20, pancreatic cancer 21, osteosarcoma 22 and hepatic metastasis of colorectal cancer 19.

### Hepatocellular carcinoma and hepatic metastasis of colorectal cancer

Panzitt and colleagues generated an HCC‐specific gene library to screen for deregulated genes using 46 HCC, 4 focal nodular hyperplasia, 7 cirrhosis and 2 non‐neoplastic liver samples. They found that HULC was progressively up‐regulated from cirrhosis, through focal nodular hyperplasia, to HCC. The overexpression of HULC was also confirmed by radioactive *in situ* hybridization 18. In another study, Wang and colleagues verified the up‐regulation of HULC in HCC using 14 pairs of tumour and para‐tumour tissues by real‐time reverse transcription (RT)‐PCR. They also demonstrated the higher expression of HULC in seven HCC cell lines as compared with the two normal human liver cell lines QSG‐7701 and HL‐7702 25. The up‐regulation of HULC in HCC has been further verified by other studies 26, 27, 28. In contrast with HCC, Matouk and colleagues demonstrated that no HULC expression could be detected in primary colorectal tumours or tumour‐adjacent tissues 19. Surprisingly, they found that colorectal cancer with hepatic metastasis, but not lymph nodes metastasis, exhibited a significant up‐regulation of HULC. However, whether hepatic microenvironment drives the overexpression of HULC or HULC by itself could promote liver metastasis of colorectal cancer remains to be ascertained.

### Gastric cancer, pancreatic cancer and osteosarcoma

By real‐time RT‐PCR, Zhao and colleagues quantified HULC expression in 58 pairs of gastric cancer and paired adjacent tissues and found that HULC levels were markedly up‐regulated in cancerous gastric tissues. They also reported that HULC expression was higher in three gastric cancer cell lines (SGC7901, BGC823 and AGS) as compared with the human gastric epithelial mucosa cell line GES‐1 20. Similar to HCC and gastric cancer, Peng and colleagues showed a significant increase in HULC level in pancreatic cancer as compared with adjacent normal tissues. The authors also demosntrated higher levels of HULC in a panel of pancreatic cancer cell lines (MIAPaca‐2, CFPAC‐1, PANC‐1, AsPC‐1, SW1990 and BxPC‐3) relative to normal human pancreas tissues 21. A recent study by Sun and colleagues further demonstrated higher HULC expression in human osteosarcoma tissues relative to adjacent non‐tumour tissues. In addition, they reported that HULC expression was significantly higher in three osteosarcoma cell lines (MG‐63, U2OS and SAOS‐2) as compared with the human normal bone cell line hFOB 22.

## Transcriptional regulation of HULC by hepatitis B virus and other factors

**Figure 1:**
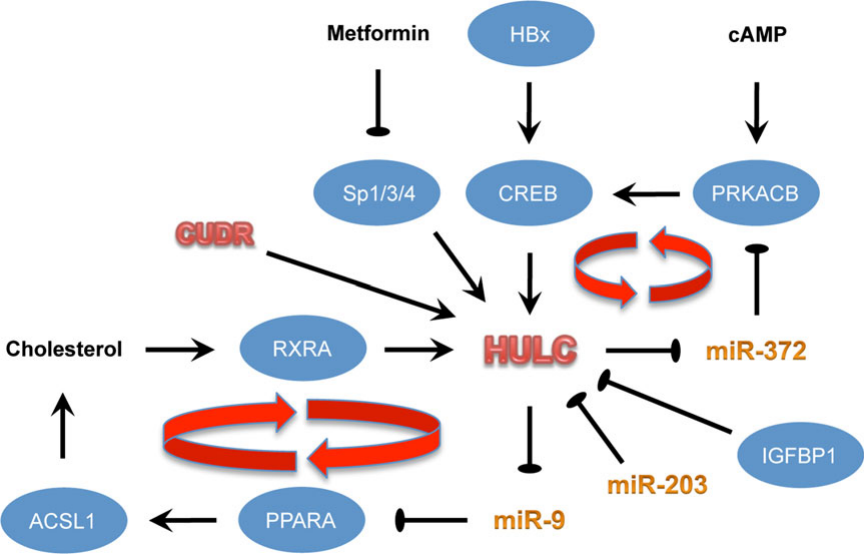
Upstream regulatory mechanisms governing HULC expression. HBx‐induced activation of CREB plays a key role in aberrant up‐regulation of HULC in HCC. Unchecked activation of two feed‐forward loops, namely miR‐372/PRKACB/CREB and miR‐9/PPARA/ACSL1/cholesterol/RXRA, also maintain HULC overexpression. Transcriptional regulation of HULC by transcription factors Sp1/3/4 and the lncRNA CUDR and post‐transcriptional repression by IGFBP1 and miR‐203 have also been reported.

Although the mechanisms underlying HULC overexpression in many cancer types remain uncertain, emerging evidence have hinted at complex interplay between environmental and host factors in the regulation of HULC expression (Fig. 1).

### Hepatitis B virus and CREB

Matouk and colleagues showed that HULC was up‐regulated in two hepatitis B virus (HBV)‐producing HCC cell lines compared with their parental lines that do not produce HBV 19, implicating that HULC might be induced by HBV during hepatocarcinogenesis. Concordantly, Lu and colleagues found that HULC levels were strongly associated with HBV X protein (HBx), an oncogenic viral protein that mediates many aspects of HBV pathogenicity, in both HCC and non‐tumourous liver tissues 19. In this respect, HBx induced the promoter activity of HULC *via* the transcription factor CREB 26, which in combination with its partner P300 triggers promoter acetylation and demethylation 25. Wang and colleagues further identified a regulatory loop between HULC and CREB, in which the former could ‘sponge’ and down‐regulate miR‐372, thereby derepressing PRKACB (a catalytic subunit of cAMP‐dependent protein kinase), which in turn induces phosphorylation and activation of CREB 25.

### Other transcription factors

In addition to CREB, other transcription factors have been identified to link environmental stimulation to aberrant HULC up‐regulation. Cui and colleagues 23 found that cholesterol could up‐regulate HULC expression through RXRA, a nuclear retinoid receptor with ligand‐dependent transcriptional activity in HCC cells. Importantly, a feed‐forward loop exists between cholesterol and HULC in which the latter could elicit methylation of CpG islands in the miR‐9 promoter and thereby abrogating miR‐9‐mediated repression of the transcription factor PPARA. Derepression of PPARA in turn drives the expression of acyl‐CoA synthetase subunit ACSL1 that catalyses the initial step in cellular long‐chain fatty acid metabolism. Besides RXRA, several members of the transcription factor Sp family (i.e. Sp1, Sp3 and Sp4) were found to positively regulate HULC expression through direct binding to *HULC* promoter in HCC cell lines. In this regard, the antidiabetic drug metformin down‐regulated these Sp proteins and decreased HULC expression 29.

### LncRNA CUDR

LncRNA cancer up‐regulated drug‐resistant (CUDR) gene is overexpressed in many tumours and could promote oncogenesis. Gui and colleagues showed that CUDR induced HULC expression *via* inhibiting *HULC* promoter methylation during malignant transformation of embryonic stem cell‐derived hepatocyte‐like cells 30. This study highlighted the complexity of gene regulation by demonstrating an unanticipated lncRNA–lncRNA interaction.

### Post‐transcriptional regulators

Hämmerle and colleagues demonstrated that HULC could be regulated by post‐transcriptional destabilization through binding to IGF2 mRNA‐binding protein 1 (IGFBP1). Mechanistically, binding of IGFBP1 reduced the half‐life and steady‐state expression levels of HULC through recruiting the CNOT1 protein, which is the scaffold of the human CCR4‐NOT deadenylase complex. These findings suggested that IGF2BP1 might induce HULC degradation through promoting HULC deadenylation 27. Apart from RNA destabilization, post‐transcriptional regulation of HULC by miR‐203 has been reported 31.

## Oncogenic functions and mechanisms of HULC

**Figure 2:**
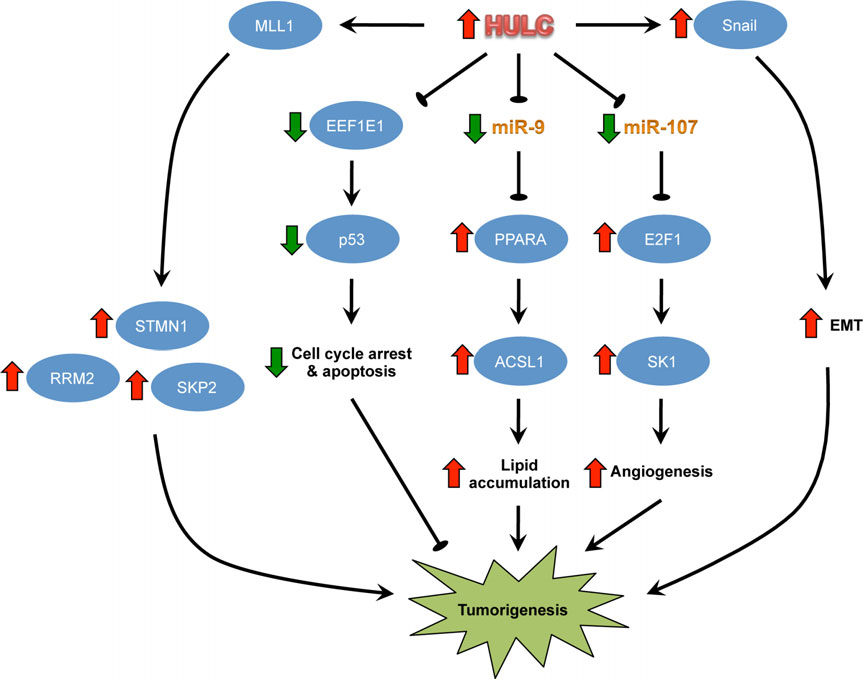
Downstream oncogenic pathways activated by HULC. Induction of these pathways by HULC was mainly reported in HCC studies. The protumorigenic mechanism of HULC overexpression in other cancer types is still largely uncertain. EMT, epithelial–mesenchymal transition.

Highly up‐regulated in liver cancer has been shown to exert oncogenic functions through promoting cancer‐related phenotypes, such as cell survival, proliferation, colony formation, migration, invasion, tumorigenicity and/or angiogenesis, in different cancer types (Table 1). The mechanism by which HULC mediates such actions is complex and involves multiple factors (Fig. 2).

**Table 1:** Oncogenic functions of HULC in human cancers. EMT, epithelial‐to‐mesenchymal transition

**Cancer types**	**Phenotypes affected**	**Regulation**	**Cell lines used**	**Approach**
HCC	Cell proliferation	Positive	MHCC97L, HepG2	Gain‐of‐function
			LO2	Gain‐of‐function
			LO2‐X, Hep3B, PLC/PRF/5, HepG2‐X	Loss‐of‐function
	Cell proliferation, soft‐agar colony formation	Positive	Embryonic stem cell‐derived hepatocyte‐like cells	Loss‐of‐function
	Cell proliferation, G_1_‐S transition, colony formation, tumorigenicity	Positive	HepG2	Gain‐of‐function
	Colony formation	Positive	HepG2‐X	Loss‐of‐function
	Soft‐agar colony formation	Positive	LO2	Gain‐of‐function
	Migration, invasion	Positive	HepG2, SNU‐449, SK‐Hep‐1	Loss‐of‐function
	EMT	Positive	SK‐Hep‐1	Loss‐of‐function
	Tumorigenicity	Positive	HepG2‐X	Loss‐of‐function
	Tumorigenicity, lipogenesis	Positive	HepG2, Huh7	Gain‐of‐function
	Tumorigenicity, angiogenesis	Positive	HepG2, Huh7	Gain‐of‐function
	Lipogenesis	Positive	HepG2.2.15	Loss‐of‐function
Gastric cancer	Cell proliferation	Positive	SGC7901	Gain‐of‐function, loss‐of‐function
	Migration, invasion, EMT	Positive	SGC7901	Loss‐of‐function
	Apoptosis	Negative	SGC7901	Gain‐of‐function
Osteosarcoma	Cell proliferation, migration, invasion	Positive	U2OS	Loss‐of‐function
Pancreatic cancer	Cell proliferation, colony formation, G_1_‐S transition	Positive	MIAPaca‐2, CFPAC‐1	Loss‐of‐function

### Down‐regulation of EEF1E1

Eukaryotic translation elongation factor 1 epsilon 1 (EEF1E1), also known as AIMP3 and p18, is a scaffold of the macromolecular aminoacyl‐tRNA synthase complex and may function as a tumour suppressor by translocating into the nucleus upon DNA damage to mediate ATM/ATR‐mediated p53 activation 32. Loss of EEF1E1 expression has been documented in gastric, colorectal and bladder cancers 33, 34. *EEF1E1* gene is in close proximity to *HULC* gene. In this connection, lncRNAs may have a propensity for regulating the expression of their neighbouring genes 35. Du and colleagues demonstrated that there was a negative correlation between the levels of HULC and EEF1E1 in HCC tissue specimens. Enforced expression of HULC decreased while knockdown of HULC increased the promoter activity and expression of EEF1E1. Importantly, abrogating the up‐regulation of EEF1E1 rescued the tumour‐suppressing effect of HULC knockdown, which *per se* was sufficient to promote HCC growth *in vivo* 26. These findings suggested that HULC promotes HCC growth at least partly through down‐regulating EEF1E1. The regulation of EEF1E1 by HULC has also been demonstrated during regulatory T‐cell differentiation in HBV‐related liver cirrhosis 36.

### Promoting angiogenesis *via* sphingosine kinase 1

Sphingolipids are important bioactive molecules that signal cell proliferation. Accumulating evidence suggests that regulation of sphingolipid levels by sphingosine kinase 1 (SK1) plays a crucial role in carcinogenesis 37. Lu and colleagues found that HULC levels were positively correlated with levels of SK1 and its product, sphingosine‐1‐phosphate, in HCC. Importantly, knockdown of SK1 abrogated HULC‐enhanced angiogenesis. The authors further demonstrated that sequestration of miR‐107 by HULC derepressed E2F1, thereby enhancing SK1 transcription 24.

### Promoting abnormal lipid metabolism by ACSL1

As mentioned above, ACSL1 is an enzyme crucial for initiating long‐chain fatty acid metabolism. Cui and colleagues reported that HULC levels were positively correlated with ACSL1 levels in HCC, in which epigenetic silencing of miR‐9 by HULC derepressed the transcriptional factor PPARA, thereby inducing ACSL1. Activation of this molecular circuitry led to the accumulation of intracellular triglycerides and cholesterol. In this connection, knockdown of ACSL1 reduced the levels of triglycerides and cholesterol and the growth of HCC xenografts in nude mice. Restored expression of miR‐9, knockdown of PPARA or ACSL1 or pharmacological inhibition of ACSL1 by Triacsin C also rectified lipid accumulation and abrogated the mitogenic effect of HULC overexpression *in vitro* 23.

### Perturbing cellular circadian rhythm *via* CLOCK

Disruption of cellular circadian rhythm (i.e. periodic alterations of gene expression) is implicated in hepatocarcinogenesis 38. It has been demonstrated that HULC could up‐regulate the circadian regulator CLOCK and perturb its rhythmical expression in HCC *via* interacting with the 5′UTR of CLOCK mRNA through complementary base pairing. Concordantly, CLOCK was up‐regulated in HCC tissues and correlated with HULC levels. To this end, knockdown of CLOCK abolished the stimulatory effects of HULC overexpression on cell proliferation, G_1_‐S phase transition and colony formation *in vitro* as well as HCC xenograft growth *in vivo* 39.

### Promoting epithelial–mesenchymal transition *via* Snail

Epithelial–mesenchymal transition (EMT) is a process by which epithelial cells acquire mesenchymal properties characterized by reduced intercellular adhesion and elevated motility and invasiveness. EMT plays a key role in tumour progression and metastasis 40. Positive regulation of EMT, manifested as down‐regulation of epithelial markers (e.g. E‐cadherin) and up‐regulation of mesenchymal markers (e.g. vimentin), by HULC has been demonstrated in HCC 29 and gastric cancer 20. In this respect, overexpression of HULC has been shown to up‐regulate the expression of Snail 41, which is an important EMT‐inducing transcription factor 42.

### Regulation of other key oncogenes and tumour suppressor genes

LncRNAs could interact with chromatin‐modifying complexes, such as EZH2 and MLL1, to regulate gene expression. Gandhy and colleagues reported that a substantial number of genes were co‐regulated by HULC and MLL1 but not EZH2 29. In particular, several key oncogenes in hepatocarcinogenesis, such as ribonucleotide reductase M2 43, Skp2 44 and Stathmin1 45, were positively regulated by both HULC and MLL1 in HCC cells. Apart from positive regulation of oncogenes, repression of tumour suppressor genes, GLTSCR2 46 and miR‐372 47, by HULC has been reported 18. However, whether these genes are functionally involved in the oncogenic action of HULC remains unclear.

## Clinical utilities of HULC

### Polymorphism of HULC gene as cancer susceptibility marker

Liu and colleagues conducted a case–control study and genotyped a single‐nucleotide polymorphism (SNP) rs7763881 in *HULC* in a Chinese cohort of 1300 HBV‐positive HCC patients, 1344 HBV persistent carriers and 1344 participants with HBV natural clearance. The authors found that AC and CC genotypes of rs7763881 conferred a significantly lower risk (*P* = 0.022) for HCC with an odds ratio of 0.81 in a dominant genetic model as compared with the AA genotype. However, no significant association was found between rs7763881 genotypes and HBV clearance 48. Similarly, in a case–control study for assessing the association between rs7763881 genotypes and susceptibility to oesophageal squamous cell carcinoma, AC genotype was associated with a significantly reduced disease risk (*P* = 0.031) relative to the AA genotype with an adjusted odds ratio of 0.69 49. These findings indicated that genetic variants of *HULC* reduce the susceptibilities to HBV‐associated HCC and oesophageal squamous cell carcinoma.

### Circulating HULC as diagnostic marker

Highly up‐regulated in liver cancerwas detected with higher frequency in the plasma of HCC patients compared to healthy controls (63% *versus* 10%) with higher detection rates in patients with higher Edmondson grades (100% in Stage III/IV *versus* 14% in Stage I/II) or with HBV‐positive status (90% *versus* 25%) 28. The diagnostic significance of circulating HULC was verified in a subsequent study, in which HULC could achieve an area under the receiver operating characteristic curve of 0.78 for diagnosing HCC. There was also a strong correlation between tissue and circulating levels of HULC 50. These findings suggested that circulating HULC might be used as a non‐invasive biomarker for HCC diagnosis.

### HULC as prognostic marker

Up‐regulation of HULC was associated with poor pathological and clinical outcome in osteosarcoma, pancreatic cancer and gastric cancer. In osteosarcoma, higher expression of HULC was correlated with more advanced clinical stages and distant metastasis as well as shorter overall survival. Multivariate analysis confirmed HULC overexpression to be an independent prognostic factor for patients’ survival 22. Similarly, higher HULC expression was associated with larger tumour size, lymph node metastasis and vascular invasion in pancreatic cancer and served as an independent prognosticator for shorter overall survival 21. In gastric cancer, HULC overexpression was correlated with lymph node metastasis, distant metastasis and advanced tumour‐node‐metastasis (TNM) stages 20.

While HULC is highly up‐regulated in HCC, its association with clinicopathological features remains controversial. Hämmerle and colleagues found that HULC up‐regulation was most prominent in low‐stage HCC and progressively decreased along advancing tumour stages. Highly up‐regulated in liver cancer up‐regulation was also more remarkable in well‐differentiated than poorly differentiated HCC 27. On the contrary, Xie and colleagues reported that higher HULC expression was positively associated with Edmondson histological grades of HCC 27. Consistent with the former, Yang and colleagues demonstrated that high HULC expression was associated with less vascular invasion and better overall survival of HCC patients 51. Further studies with larger sample size are needed to ascertain the prognostic significance of HULC in HCC.

## Concluding remarks and future perspectives

Overexpression of the lncRNA HULC occurs in many cancer types, including hepatocellular carcinoma, gastric cancer, pancreatic cancer, osteosarcoma and hepatic metastasis of colorectal cancer. A complex interplay between environmental factors (e.g. HBV infection, cholesterol) and existing host cellular signalling dysregulation (e.g. transcription factors, miRNAs) might contribute to its aberrant up‐regulation. As an oncogene, HULC promotes cancer‐related cellular phenotypes *via* multiple pathways, which further our understanding of the complexity of gene regulation by lncRNAs. However, these pathways interact widely with each other and their significance in the oncogenic action of HULC should be interpreted with caution. From a clinical perspective, polymorphisms in *HULC* gene are associated with altered risks for oesophageal cancer and HCC, whereas HULC in plasma may serve as a biomarker for early HCC diagnosis. Moreover, altered expression of HULC has been shown to correlate with clinicopathological features, including patients’ survival. Nevertheless, population‐based differences may occur, and thus its use as a biomarker should be verified in different ethnic groups. Despite these limitations, it is propitious that our understanding of the upstream regulatory mechanisms of HULC and recent advances in the development of RNA‐targeting therapeutics will eventually open up new avenues for developing HULC‐targeting molecules as novel cancer therapeutics.
